# Public health approach of clinical trials: Cuban’s experience of research translation into clinical practice

**DOI:** 10.1186/1472-6963-14-S2-P149

**Published:** 2014-07-07

**Authors:** Maria A Pascual, Martha M Fors, Gladys Jiménez, Isabel López, Ania Torres

**Affiliations:** 1 National Centre of Clinical Trials (CENCEC) Havana, 11600, Cuba

## Background

Controlled Clinical Trials are the Gold Standard to assess the efficacy and safety of a new therapeutic intervention. Furthermore, their results are used to make decisions in relation to public health issues[1]. This study describes the experience of the National Centre of Clinical Trials (CENCEC) of Cuba[2] introducing research results into medical practice, extending new therapeutic technologies, identifying barriers to the translation of research into medical practice, improving the evidence for making decisions in public health and improving the delivery of medical services[3].

## Material and methods

All trials led by CENCEC from 1992 to 2013 were characterized according to specific variables. A review of available information at the Cuban Regulatory Agency regarding the registration process of new drugs was performed. Interviews to sponsors, clinical investigators and health authorities were carried out to identify products registered outside the country, health benefits and impact of the results of clinical trials conducted by CENCEC.

## Results

In this period, 133 clinical trials were completed evaluating 58 products from 28 sponsors, with participation of 1075 clinical sites from 90 hospitals and 60 primary care health care units involving 4241 researchers. Some of those studies were performed for the evaluation of health technologies and to search evidence for public health decisions. The activities of CENCEC were carried out according to international standards: Quality Assurance System Certificated (ISO 9001:2008), Cuban Public Registry of Clinical Trials (WHO Primary registry [4] and role as regional coordinator of Good Clinical Practices Working Group (PANDRHA) among others.

The benefits of "new intervention" clinical trials were related to morbidity and mortality rates, new patterns of disease management, better Infrastructure of clinical sites, improvement of the quality of medical care, the introduction of new technologies and the building of capacity of clinical investigators [5].

## Conclusions

The experience of Cuba, a low income country with a defined health policy, shows that the results of clinical trials are an effective tool to improve health services and for an efficient introduction of evidence in medical practice, for decision making in public health leading to improvements in clinical care. The experience gained could be applied in other countries of Latin America to achieve public health results .
